# 2117. *In Vitro* Antimicrobial Activity of Taurolidine against *Candida auris* Bloodstream Isolates from Global Sources

**DOI:** 10.1093/ofid/ofad500.1740

**Published:** 2023-11-27

**Authors:** Anthony Pfaffle, Leonard R Duncan, Mariana Castanheira, Bruce Reidenberg, Cecilia G Carvalhaes, Jessica Vaughn, Phoebe Mounts

**Affiliations:** CorMedix Inc., Berkeley Heights, New Jersey; JMI Laboratories, North Liberty, Iowa; JMI Laboratories, North Liberty, Iowa; Independent (on behalf of CorMedix), New York City, New York; JMI Laboratories, North Liberty, Iowa; Wiley Rein LLP (formerly Cormedix Inc. May 2019-Jan 2022), Washington DC, District of Columbia; CorMedix Inc., Berkeley Heights, New Jersey

## Abstract

**Background:**

Taurolidine (TAU) exhibits broad antimicrobial activity and is under development as a component of a new catheter lock solution with the goal of reducing catheter-related bloodstream infections (CRBSI). The Centers for Disease Control and Prevention (CDC) have recently warned that the fungus *Candida auris* (CA) is an emerging pathogen of concern, and surveillance studies have shown an increase in the number of BSI caused by CA over the last 3 years. In this study, the *in vitro* antimicrobial activity of TAU was evaluated against a set of CA strains/isolates using reference methods.

**Methods:**

41 CA strains/isolates were obtained from the CDC Antimicrobial Resistance Isolate Bank, the Westerdijk Fungal Biodiversity Institute, and JMI Laboratories’ SENTRY Antimicrobial Surveillance Program. Most CA isolates were from BSI. The CA set was tested for antifungal susceptibility using Clinical and Laboratory Standards Institute (CLSI) broth microdilution guidelines and supplemented Roswell Park Memorial Institute 1640 broth. JMI Laboratories produced the minimal inhibitory concentration (MIC) panels. CLSI-recommended quality control strains were also tested. MIC values were read after 24 hours. TAU MIC values were read at 50% and 100% growth inhibition. Tentative CDC breakpoints for amphotericin B and fluconazole were applied.

**Results:**

The full set of CA isolates was 53.7% resistant to amphotericin B and 85.4% resistant to fluconazole. TAU exhibited antimicrobial activity against all CA strains and isolates regardless of source, clade, or known resistance mechanisms (MIC_50/90_ values, 256/512 mg/L using the 50% inhibition reading criterion and 512/512 mg/L using the 100% inhibition reading criterion). The TAU MIC range was 128–512 mg/L and 256–1,024 mg/L using the 50% and 100% inhibition reading criteria, respectively. Additional CA isolates are being tested.

**Conclusion:**

TAU activity was similar for all CA subsets tested (overall MIC_50/90_ values, 256/512 mg/L using the 50% inhibition reading criterion). There was no evidence that TAU activity was affected by isolate source or clade. Based on these data, catheter lock solutions containing the broad-spectrum antimicrobial TAU at 13,500 mg/L have the potential to prevent CRBSI caused by CA.

**Disclosures:**

**Anthony Pfaffle, MD**, CorMedix: Salary|CorMedix: Stocks/Bonds **Leonard R. Duncan, PhD**, AbbVie: Grant/Research Support|Basilea: Grant/Research Support|CorMedix: Grant/Research Support|Melinta: Grant/Research Support|Pfizer: Grant/Research Support **Mariana Castanheira, PhD**, AbbVie: Grant/Research Support|Basilea: Grant/Research Support|bioMerieux: Grant/Research Support|Cipla: Grant/Research Support|CorMedix: Grant/Research Support|Entasis: Grant/Research Support|Melinta: Grant/Research Support|Paratek: Grant/Research Support|Pfizer: Grant/Research Support|Shionogi: Grant/Research Support **Bruce Reidenberg, MD, FAAP, FCP**, CorMedix: Advisor/Consultant **Cecilia G. Carvalhaes, MD, PhD**, AbbVie: Grant/Research Support|bioMerieux: Grant/Research Support|Cipla: Grant/Research Support|CorMedix: Grant/Research Support|Melinta: Grant/Research Support|Pfizer: Grant/Research Support **Jessica Vaughn, JD, PhD**, CorMedix: Former employee (no stock or stock options) **Phoebe Mounts, JD**, CorMedix: Slary|CorMedix: Stocks/Bonds

